# Attitudes of blood donors to their sample and data donation for biobanking

**DOI:** 10.1038/s41431-019-0434-1

**Published:** 2019-05-30

**Authors:** Vera Raivola, Karoliina Snell, Ilpo Helén, Jukka Partanen

**Affiliations:** 1 Finnish Red Cross Blood Service, Helsinki, Finland; 2 Helsinki Collegium for Advanced Studies, University of Helsinki, Helsinki, Finland; 3 Department of Social Sciences, University of Eastern Finland, Helsinki, Finland

**Keywords:** Ethics, Human behaviour

## Abstract

Modern biomedical and genetic studies require large study cohorts; blood donors have been suggested to represent an appropriate group for recruiting healthy cohorts. The Blood Service Biobank (BSB) in Finland was recently established to recruit blood donors willing to give broad biobank consent. The aim of the present study is to understand how the blood bank context influences views on donating samples and health data. We organised 61 interviews and 10 group discussions with current and potential blood donors. Using qualitative content analysis, we identified three discussion frameworks that summarise the results. We found that frequent blood donors associated the voluntary act of donation with caring for patients. The blood donation experience was considered to accommodate biobank participation, but also allowed critical observations on the integration of research data collection into blood donation. Research participants identified an important difference between the blood bank and biobank contexts. In the biobank context, the focus shifts from donating blood to patients into donating personal and genetic data for research use. Blood donors’ anxiety over data use was balanced with their experience of the trustworthiness of the Blood Service. These experiences indicated that the new biobanking activity could be trusted to a familiar organisation. To build donors’ trust, biobanks should invest in their institutional reputation, donor experience and dialogue with donors. These findings can be applied to other institutions that are considering setting up biobanks with broad consent for personal data use.

## Introduction

Modern biomedical and genetic studies require large study cohorts that may be collected in biobanks with broad consent for sample and data use. Biobanks have different forms not only depending on the samples and data they collect, but also regarding their institutional context and recruitment of donors. Hospital biobanks primarily collect samples and health records from their patients, whereas population or epidemiological cohorts aim to collect representative samples of the entire population. It has been suggested, and there are good examples to support this, that blood donors would constitute a good cohort representing healthy populations [1]. Integration of a biobank into a traditional blood bank has advantages. The existing blood collection infrastructure and pool of blood donors can be effectively utilised for research purposes. For researchers, a blood donor biobank offers access to a longitudinal collection of samples in addition to genetic and health data [2]. Blood donors have, furthermore, been reported to have higher than average willingness to participate in biobanks [3] and to approve genetic research [4]. Indeed, some blood donor biobanks have been very successful in recruiting blood donors [2, 5].

To understand why this is so, we studied the views of current blood donors and potential donors on biobanking and use of their samples and data for research and analysed how they regarded such activities in comparison with the standard blood donation to patients. Our aim was to understand whether the blood bank context has particular characteristics related to biobank participation. The biobank in the present study was the Blood Service Biobank (BSB), operated as part of the Finnish Red Cross (FRC) Blood Service, the only blood establishment in Finland. At the time of the collection of focus group data in spring 2017, the FRC Blood Service was planning to establish its own biobank, a plan which was implemented in November 2017.

Biobank activities in Finland are regulated by the Biobank Act (688/2012). The Act provides a framework for the utilisation of human biological samples and data in future medical research and product development, while securing rights of biobank research participants [6]. The Act defines criteria of a biobank and states that all biobanks must be registered. To register a biobank, the organisation must have permits from the ethical evaluation authority (TUKIJA) and from the National Supervisory Authority (Valvira) [7]. Currently, the BSB is one of the 10 registered biobanks in Finland. The national regulation dictates the basis for biobanking practices [7]. According to the Act, all biobank research is based on broad consent. The Act gives biobank participants the right to withdraw their consent and to request from the biobank information on the use of their samples and the clinical significance of obtained health information. The use of information is limited, excluding certain purposes and de-identification policies are imposed [6]. With the encompassing national biobank regulation in place, the May 2018 General Data Protection Regulation (GDPR) has so far not resulted on changes for biobanking and participant rights. For example, the BSB need not change the information on biobank consent for blood donors after the GDPR. Surveys from Finland report a comparatively relaxed public attitude to the broad consent [8] and positive outlook on biobanks [9]. A recent survey, however, found that majority felt their knowledge on biobanks was still insufficient [10]. The Blood Service Biobank began recruiting blood donors for biobanking at blood donation sites on November 2017; all focus groups took place before the BSB launch. Nurses inform blood donors of the opportunity to give the broad consent and an aliquot of blood for research. In our previous study [11], we found that Finnish blood donors were willing to donate blood also to scientific research in addition to a standard blood donation. Contributing to research was considered to align with blood donors’ motives of helping patients and contributing to common good. For the interviewees, the concrete link to patients who need blood and the ease of familiar blood donation practice was important.

In the present study, our focus is on understanding how current and potential blood donors perceive the BSB in the context of blood donation. This study was done before the launch of the BSB, so our questions are prospective in nature. Previous studies on biobank participation show these decisions to be very much dependent on donor’s perception of the social context [12]. How a potential biobank participant interprets this context—i.e., who is asking for donation and how, and what are the institutions and risks involved—can influence the decision more than formal legal and technical information on biobanks [8, 13, 14]. In our case, biobank participation was assumed to take place in the context of the FRC Blood Service that has a long tradition in blood procurement from voluntary donors. A success of any blood bank depends not only on its physical infrastructure but also how well the message it promotes facilitates donations [15]. The BSB’s slogan “A new dimension to extend your help” [16] can be seen as an example of such message that promotes a donor perception in which donating blood to biobank represents a continuum of standard blood donation. Considering the increasingly complex systems procuring blood, deciding for or against different donation purpose can be challenging [17]. Use of qualitative methods provided us with a valuable means of analysing what arguments make participation sensible and appropriate from blood donors point of view. We see that the findings of this analysis have a more general application to the biobanks.

## Participants and methods

### Recruitment

Our research data consist of 61 blood donor interviews ranging from 10 to 30 min and 10 focus groups discussions that were approximately 1.5 h long each. The interviewees were organised at seven different blood donation sites to capture a sufficient range of viewpoints. More information on the interviewees and interview recruitment processes is provided in Supplementary material (Tables S1 and S2). Ten focus groups (Table 1) were formed from university science students (FG 1–2), Finnish Defence Force privates (FG 3–4), frequent blood donors (FG 5–6) and participants of the GeneRISK study (FG 7–10). The organisation of the focus groups was based on the following objectives: groups that represent different stages of experience on blood donation (none—frequent) with focus on younger age groups and on blood donors who already had participated in a genetic study through the Blood Service (GeneRISK). We wanted to include a group of non-blood donors and mixed groups of donors and non-donors to the data composed of discussions with frequent blood donors. The idea was to observe (1) what views were generally shared among all participants; and (2) how practical experience from the Blood Service was reflected on views. The GeneRISK study was conducted in the Blood Service among interested blood donors from the age group of 45–60. The GeneRISK study subjects (www.generisk.fi) received information on their genetic and lifestyle related risks of cardiovascular diseases. These participants are different from the other groups as they had signed a consent for the THL Biobank as part of the GeneRISK study protocol.

**Table 1:** Focus group participant information

Focus group (ID)	Members (*n*)	Men (*n*)	Women (*n*)	Average age	Have donated blood (%)
Helsinki University students (FG1)	6	2	4	21	0
Helsinki University students (FG2)	7	3	4	21	100
The Finnish Defence Force privates (FG3)	7	7	0	20	29
The Finnish Defence Force privates (FG4)	8	6	2	21	50
Frequent blood donors^a^ (FG5)	6	3	3	40	100
Frequent blood donors^a^ (FG6)	9	5	4	48	100
Blood donors in GeneRISK study (FG7)	9	3	6	57	100
Blood donors in GeneRISK study (FG8)	6	4	2	58	83
Blood donors in GeneRISK study (FG9)	4	2	2	56	100
Blood donors in GeneRISK study (FG10)	6	4	2	63	100
Total	68	39	29	41	76

^a^Whole blood donation times ≥7

### Data collection

Open-ended interviews were conducted by V.R. and the discussions groups were moderated by V.R. or K.S. Questions for both interviews and focus groups covered four themes in the following sequence: (A) donating blood for patients, (B) donating blood and information for research and (C) participation to the BSB, and (D) the possibility to return genetic risk or health data to donors. Description of interview questions topics can be found in Supplement S3. Data collection continued until the accumulation of viewpoints was observed to reach saturation, thereafter additional interviews were likely to have only a modest increase in the qualitative variety of the data, i.e., topics raised. All discussions were recorded. The two methods of data collection were combined to capture a broader variance in content and reasoning. The blood donor interviews provided a more structured outlook than the range of available accounts. Focus groups as moderated discussions can illuminate how social and cultural understanding of a specific topic evolves through interaction [18].

### Data analysis

The discussions in the interview and focus groups were transcribed verbatim and anonymized; interview citations (I) show the interview number and respondents age/gender and examples from focus group discussion (FG) are referred to only by group/line numbers to protect the identity of speakers. Transcripts were uploaded into Atlas.ti (version 7.0; Scientific Software Development GmbH, Berlin, Germany), a qualitative data management and analysis software. The analysis evolved inductively following the basic principles of the qualitative content analysis [19]. First, a descriptive coding frame was developed. Coded segments related to the BSB were compiled in a matrix and then categorised under themes. The second order coding was an iterative process to control consistency within and between the texts. Finally, themes were compared with the research questions to bring together a valid empirical summary of key perspectives.

### Ethics

The interviews and focus groups were organised in compliance with the Finnish National Board of Research Integrity principles of ethical review for human sciences [20]. Since this study did not expose participants to any major risks or harm, it was considered not to require a formal ethics approval. Recruitment was designed to ensure that the participants of the study were properly informed and participated voluntarily. Interviews were conducted anonymously. Focus group members signed a consent and were rewarded with two movie tickets.

## Results

### Blood bank vs. biobank

The majority of the participants reported having come across term biobank but described feeling uncertain of its exact content. Therefore, to start conversations, we gave everyone a short summary about biobanks in Finland according to the Biobank Act, including broad consent, and had a general discussion on their perception of biobank research. Then we enquired how participants felt about the plan to set up the Blood Service Biobank (BSB) and if they, as blood donors, would be willing to join the BSB by giving a blood sample for this purpose.

Apart from very few exceptions, the participants held a positive attitude towards joining the BSB (Table 2: Quotes 1–2). They saw no problem in combining blood donation with biobank collection to advance medical research for better treatments. Giving a blood sample also for the BSB seemed: “[…] on the first reflection, to fall in the same category as blood donation” (FG4: 329–331), constituting a sensible personal investment in improving public health care. The BSB was regarded as offering donors an additional opportunity to help others, since contributions to medical research could benefit social wellbeing. The cost and risks of giving an extra sample of blood seemed relatively low to blood donors. From a wider perspective, the FRC Blood Service was thought to hold the capacity to collect genetic and other health research material. (Table 2: Quotes 3–5).

**Table 2:** Quotes illustrating theme "Blood bank vs. biobank"

Quote number	Interview (I) ID/Focus group (FG) ID	Citatations from study participants (P)
1	I:22M47	P: Well, it’s fine with me. It is like if you are ready to have an organ-donor card or something like that, it is not any more strange idea to have a drop of blood stashed somewhere, all the same
2	I:7F50	P: I think that it is a good thing; I don’t find it threatening or something that could be misused. At least I believe that it would be quite a good thing
3	I:15M25	P: That seems quite practical, it would be easy to collect it there. Likely many people would donate for it, so that there could be a good amount of data in safe
4	FG6: 457–471	P: So if those biobanks already exist, why make one more? Somehow I feel that if all that is known about me would be there in one pile in some cloud, so then if someone who needs that information then they would get it from there. Whether that would be FRC [the Finnish Red Cross, VR] or health care services or who damn ever, I would think it would be clearer than having a biobank here and there, and little bit of this information about me here, and then some other thing there
5	I38F28	P: It [the Biobank, VR] sounds modern, I mean good because when there is system like that when totally new diseases appear then there is some material to at least start researching
6	FG2: 444–448	P1: If many individual things about me are then put together, it comes to mind, that it becomes more possible that someone might use that for their benefit. Something that I did not intend when I have wanted to take part, wanted to help in some scientific research P2: What does it mean, that some random researcher somewhere knows more about your life than you do, like, know that you are going to buy food X or to do this...
7	FG5: 408–414	P1: For me it is, that at least it [data, VR] is sufficiently protected, whatever that might mean. That no hacker cannot get in. But it is just that I do not understand these things P2: And then those people who operate the biobank, the staff, they need to be educated to know what can be done with it
8	I:137F21	P: Well in principle I would be prepared to give my data, but I would want to be informed each time before some research that for what purpose it’s used. That what kind of research my data is being used at. I think it sounds good that it is possible to withdraw if it feels that the research does not fit with my values somehow
9	I:51F26	P: Of course, when you say it is voluntary so that everyone can make their own decision whether to take part or not. I don’t see any bigger problem there then
10	FG1: 579–580	P: Isn’t it the basic right of any research participant to know where the sample goes, if one takes part?
11	I:39F53	P: It might be a good organisation to take care of it [the Biobank, VR]. There has been talk about resources regarding place and personnel and this eternal saving and rationalisation. So if this kind of addition might bring something that might help the basic operation to keep in better strength. So I would see that a positive thing
12	FG3: 434–440	P: It would be a shame if blood donation would become profiled as research; that’s unlikely to happen, but if it would, I would see that as a bad thing (…) Because for me, and perhaps many, the value in that activity [blood donation, VR] and why to continue is that it can really, concretely help a person. If it changes into some kind of research centre, some might lose their motivation because the focus is no longer in helping sick people and there is some research on the side. I think it would be important to keep the focus on helping patients

As the discussions progressed, participants concluded that the process of donating to the BSB was, however, not equivalent to the donation of blood for immediate needs of the patients. During the discussion, the participants often became increasingly aware of the fact that the right to access and use the donor’s genetic, health and lifestyle data, rather than the blood in itself, was the focus of the biobank collection. As donors to the BSB they would share potentially sensitive information—and in case of genetic information, data on their relatives as well—and make it available for research for an unspecified period of time and purpose. (Table 2: Quotes 5–6). Unlike blood at the blood bank that was considered only for the treatment of hospital patients, the data stored in the biobank seemed to offer multiple possible future applications. When discussing donating in the biobank context, participants had to address the challenge of identifying the future beneficiaries of research. Consideration of the uncertainty regarding biobank donations provoked both high hopes on social benefits and cautious reminders about ethical risks involved in allowing valuable data to be used for the wrong ends.

Not everyone was prepared to hand over the rights to their health information for unspecified purposes. If nothing else, the decision required the participants to re-evaluate their expectations regarding the capability of the organisation to manage donors’ data; whether or not the organisation was able to protect their identity and safeguard the data against unauthorised access. They accepted sharing their data because it would only be used for beneficial scientific projects that meet the requirements set by laws and ethical standards (Table 2: Quote 7–8), hence, transparency on the part of the BSB was also regarded as important.

Consent was often described to be an important point in the process where the voluntary donor should be made aware of these risks but also of the social value of the donation. Most of our study participants took it for granted that involving blood donors in research in which they would remain identifiable automatically required their separate consent and the right to decline and retract (Table 2: Quote 9–10). Frequent blood donors would also consider the option of stopping donating blood if the FRC Blood Service wasted or used the donations in ways that caused suffering or injustice. Knowing that human life was at stake, the participants felt confident that the FRC Blood Service strived to avoid disturbances to voluntary blood donations. They regarded failures in data management or research responsibilities on the part of the BSB as implausible. Yet, many saw research data collection as a secondary objective for the blood bank. (Table 2: Quote 11–12).

### Donation experience as a positive resource

The choice to donate data to the BSB was also discussed among the frequently donating donors as a practical matter in terms of convenience and habits. Experienced blood donors had the advantage of knowing the process for donating blood and providing health information at the Blood Service and they could therefore apply this experience to biobank participation (Table 3: Quotes 1–2). From the point of view of a frequent blood donor, the idea of integrating research data collection into blood collection appeared a convenient solution and an effective way to organise biobank participation. However, some frequent donors expressed concern about integrating voluntary blood donation and BSB recruitment. The discussion was focussed on whether the decision was too complex and personal to be made in a situation where the person’s primary purpose and focus was on blood donation (Table 3: Quote 3). However blood donation was often perceived to be a less problematic context for biobank recruitment because it is a voluntary activity (Table 3: Quote 4).

**Table 3:** Quotes illustrating theme "Donation experience as a positive resource"

Quote number	Interview (I) ID/Focus group (FG) ID	Quotes from study participants (P)
1	FG6: 397–399	P: For me it depends a lot about the type [of donation, VR]: if it is some particular day or two days a week when the Blood Service is open then I could go donate that sample, why not
2	I:31M18	P: In practise, it could be done at the same time when you are normally donating blood. That one could, some part [of blood, VR] would just go there. I don’t see any problem with that
3	FG5: 426:446	P1: Now it’s so fluent, easy that you go there, haemoglobin, hand straight and off you go. If every patient would have that explanation that would you join the biobank, what is biobank, what does it mean, that wouldn’t be practical. Plus there would be that feeling that if you don’t want to do it could be difficult to say that I just want to donate blood. Would someone think not to go donate, if every time someone asks about the biobank, I don’t know. It just isn’t very easy decision for everyone, I thin, that biobanking P2: They are two different things, after all, [blood] donation and then research P1: One blood sample, one syringe gets taken there and then forward, but it is not the sampling that is most important there, but what happens to the sample afterwards and what impact does it have P3: It might really increase the threshold for some, that biobank there, that I cannot go, takes too much time at there
4	FG6: 623–629	P1: Better be on the safer side there, so that it really is consent from the donor, so that it is not asked by passing in there, with small… P2: Yes, or it would be in small writing that if you do not deny then… P3: If you are in the hospital not in the best shape and then in those conditions you are being asked different kinds of questions then you not on your sharpest: did you really understand what was asked, what for did you give your consent

### Reputation and trustworthiness

Despite the new kinds of uncertainties with regard to the donation of biobank data, the participants were generally willing to join the BSB because they trusted the Blood Service—and the welfare institutions it served—to ensure good social outcomes and to protect donors and their data. Yet the trust was not granted without reasoning; instead participants used different indicators to estimate whether the Blood Service was trustworthy in the context of biobank donations (Table 4: Quotes1–3). The Blood Service’s public reputation appeared to be based on history of good performance. For many, being part of the Finnish Red Cross signalled accountability. A voluntary charity should guarantee stability and ensure that humanitarian values will not become subordinate to political and financial gains.

**Table 4:** Quotes illustrating theme “Reputation and trustworthiness”

Quote number	Interview (I) ID /Focus group (FG) ID	Quotes from participants (P)
1	I:11F51	P: I do trust that in country like Finland studies are made with high professional ethics and minding the patient so that never to take too much blood or any cells. I do not believe there is any risks for the research participant (…) No it came to mind all things like sleep apnea that has been caught from some pills, and (laughs). But no, I do trust that study participants are well taken care of and research is done in the limits of what people endure
2	I:14F23	P: Well that [the Blood Service, VR] must sound the most trustworthy I personally can think of. So that I would have a positive view on that [the BSB, VR]
3	FG6: 351–355	P: So if we think what kind of trust Finnish people have towards the FRC [the Finnish Red Cross, VR] and that if the FRC starts doing something like this, that immediately feels like it does not need to be questioned so that why not give [consent, VR]
4	I:35TuM27	P: Wouldn’t that be a natural extension, if those [biobanks, VR] are anyway being established that this would be straight under the Blood Service’s own control
5	FG6: 407–414	P1: This event [focus group discussion, VR] in itself is some kind of proof that whatever those blood suckers suggest, we are ready to go for it; that our trust is so strong P2: When I got this invitation, I was with my husband. We quite rarely donate together, but that was one occasion. And then when I was given this note, my husband was like “I didn’t get, why I didn’t get” P1: But also hunger takes people into many situations... P3: That’s another thing, where ever there is coffee to serve (laughs)
6	FG1:577–579	P: It would be nice to have that kind thing that if there comes a company and gives a little money and says that lets’ take a blood sample, then those who control the data sends to the person who owns the blood sample this information as would you like to give your blood to this study or will you deny it. That there would be something like that. But on the other hand it only extends these processes and then people anyway say yes or no
7	FG3:334–338	P1: Also I don’t see it to be really realistic, but if suddenly it would turn out that there is a patent owned by one company who knows how many decades and that blocks some poorer people to get the treament P2: Yeah. But I don’t see that this would be a problem about the Blood Service Biobank. That is the problem of international patenting system. That same case works anywhere. I think making information available i valuable as such, regarldes of its use. I don’t think that much harm can be made out of it. Of course if someone started developing some bioweapons that would be kind of an extreme negative case. That I would not want to happen. But I don’t see that is a realistic fear
8	FG2: 430–436	P1: Well I would began feeling anxious at the point if my personal data started appear live in some register P2: But that’s more something like Google’s rather than the Blood Service’s business
9	FG9:353–355	P: Well let’s put it in this way that I have some study background, so that the future in which this biobank data could be used for something [bad] like that is still so far away. That I don’t see that would be a reason, but of course…Like I previously said that I trust the Blood Service, that kind of retains that I would not believe that the world would change so much that the Blood Service would become an organisation that would do business with that data. Or that it would be misused or I believe that kind of future is still likely to be very far away from our lifetime

For blood donors who visited the FRC Blood Service frequently, trust was also a result of positive experiences from their encounters with the Blood Service. Nurses who organise the entire donation process with the donors had an important role in assisting, caring for and offering information to the donors. Their professional performance was central in ensuring that the blood donation was experienced as the “easy” and low-risk experience that the frequent donors described it to be. Those things that frequent blood donors saw as signs of consideration for their wellbeing and best interests, e.g., haemoglobin tests and information provision, were fundamental in building trust. They were regarded as evidence of the Blood Service’s commitment to cooperation. (Table 4: Quotes 4–5). The participants expected that the same norms of care and reciprocity would also be applied to the new form of cooperation with the BSB. However, trust was not equivalent to uncritical acceptance. A scenario that worried the participants was that the BSB intended to treat their voluntary donations as a business opportunity. By choosing commercial profit as its goal, the organisation would exploit the solidarity of blood donors. (Table 4: Quote 8–9). According to the Finnish biobank law, the biobanks must grant appropriate third parties the right to use the data in the biobank; this brought up new questions about commercialisation and data ownership. (Table 4: Quotes 6–8). Participants were not generally against involvement of the private sector where they recognised potential social benefits, but they thought that selling blood for profit or handing donor data to private ownership was against blood donors’ intent, hence, a serious breach of trust. Conversations between donors who opposed commercial involvement and those who accepted or allowed it did not always result in a consensus.

## Discussion

According to previous studies, participation in genetic research and biobanks is strongly influenced by the way a potential participant interprets the social and institutional context of the request to donate [8, 12, 14, 21]. The Europeans are likely to face a complexity of contexts and varied purposes for which donation of tissue and data are requested [17]. In the present study, we investigated views of potential and frequent blood donors on blood donation as the context for scientific research and biobanking. We collected data from 61 interviews with current blood donors and 10 discussion groups consisting of people with and without practical experience of blood donation. The aim was to see how shared understanding on voluntary blood donation as a long-standing national institution and having practical experience from the Blood Service steered their views on biobank participation. All participants were asked to reflect on the scenario in which they would be asked during ‘standard’ blood donation to give a sample and broad consent for health information to the BSB. The principles of biobank participation were explained according to the Finnish Biobank Act. We found that placing the biobank within the Blood Service was mostly considered a good idea, which made the thought of biobank participation more acceptable for those who were already blood donors. In general, being able to consider biobanking against the more familiar institutional context of blood donation helped participants to construct a more concrete view of participation. Ideas about and especially personal experience from blood donation enabled them to adapt to, but also critically evaluate, the difference between giving blood to patients and sharing personal data with research. Our study also identified three main themes in the discussions on biobank participation in the blood donation context. These were similar to those found in previous studies on willingness to take part in genetic research through biobanking: (i) belief that biobanks contribute to the public benefit [9, 22], (ii) perception of the convenience of donation [23], and (iii) trust [8, 23, 24]. With a systematic qualitative analysis, we were able to further explore the contextual meaning of these themes to blood donors and identify supporting arguments for biobank participation more generally. However, our method did not allow us to test the prevalence of the viewpoints within or between populations or to estimate how widespread they were.

We found that the experience of blood donation provided frequent donors with a framework for envisioning what biobank participation could mean in practice and in relation to the values they were volunteering for. Often formal biobank materials offer important technical and legal information on the purpose of biobanking activities but may not give straightforward answers to more pragmatic and moral questions that are of contextual nature [13, 14, 25]. These questions were at the centre of our study: What kind of experience will a biobank participation be? How to make sure that one’s contribution to biobank research will increase, not decrease, wellbeing? Using blood donation as a benchmark for biobank donation helped donors to assess whether participation in the BSB was acceptable or a good choice for them. Understanding of blood donation provided a comparative viewpoint for deliberations on such questions and also allowed constructive criticism related to the challenges of the arrangement. However, integrating biobank recruitment into the familiar blood bank context could, for some frequent donors, result in the blurring of the line between giving blood to transfusion patients and sharing personal data with researchers that could involve risks related to data control. To promote blood donors’ willingness to participate, the biobank should take seriously their wish to see a concrete link between research donations and welfare benefits for patients [11]. Lack of transparency could backfire if blood donors later felt that their goodwill or flexibility was exploited.

### Building trustworthiness

We found that frequent blood donors were mostly optimistic about the benefits of contributing data to the BSB. Cooperation with the biobank was also recognised to require trust to mitigate the uncertainty and risks involved in such a project [26]. Trust has been shown to be a requirement for donors’ willingness to take part in biobanking [23, 24]. However, trust has been proven difficult to operationalise on a practical level [26]. When exploring how blood donors came to define trustworthiness in relation to biobanking in the context of voluntary blood donation, we found three dimensions. We believe these could be of use in developing public engagement more generally also beyond this specific context.

#### Institutional trust

Biobank participation and broad consent seem to correlate positively with institutional trust [8, 21, 27]. The general public tends to view biobanks as a public good [22] and to favour governance structure independent of commercial or state control. From the viewpoint of donors, this setting offers the best way to guarantee that benefits are publicly shared [28]. For blood donors, the Blood Service provided a context in which it seemed realistic to assume that positive outcomes of the biobank donation would outweigh the risk. As a not-for-profit, well-recognised organisation serving the public health care, the Blood Service was considered to be a suitable candidate to host a biobank. The Blood Service, depending on voluntary support, seemed to anchor also the BSB into charitable values and transparency with regard to its goals. Transparency was important since some level of commercialisation seems inevitable in connection with biobanking. Commercialisation is likely to remain a contested topic among blood donors who saw that their values in volunteering conflicted with private profit gains.

#### Investing in donor experience

We found that familiarity that was based on positive experiences of the Blood Service helped frequent blood donors to accept participation in the BSB. We know from previous studies that donors often do not carefully read the full formal information about biobanking or genetic research given to them but rather rely on contextual clues of trustworthiness [14, 21]. There is also evidence that familiarity with biobanking through some previous engagement [8], and feeling comfortable with the idea of donating blood and DNA increases willingness to give broad consent to the biobank [23]. We noted how frequent blood donors evaluated trustworthiness through the practices in caring for the donors and the minimisation of risks: e.g. health tests, the expert guidance by nurses, and even the refreshments provided. The threshold for frequent donors to donate more blood or give more detailed information to the Blood Service was low when the process was perceived as a low-risk, low-cost, and rewarding way of making a positive contribution—also for research [11]. Blood services around the world invest heavily in donor satisfaction [15]. In turn, blood donor biobanks recognise that one of their main assets is the loyal base of healthy donors who are willing to come frequently to donate blood [2, 5].

#### Maintaining dialogue with donors

Both biobanks and blood banks rely on public support [15, 22]. Studies show that to gain and retain participants’ support, biobanks need to learn how best to communicate the benefits of biobank research to the community. Biobanks should also try to address people’s concerns about biobanking [27] and acknowledge their wish to participate in discussion on how their samples are used [9]. Community engagement is a dialogic process to learn about the interests of participants and to engage them in developing better practices [24]. Reasoned debate has been shown to contribute to achieving public acceptance for contested aspects of biobanking, such as commercialisation [28]. Exchanging information not only increases confidence, but reciprocity signals mutual respect between researchers and participants and valuing of donors’ contribution [11, 29]. Blood banks have systematically promoted public understanding of the benefits of blood donation and have been successful in creating culture specific discourses that resonate with moral views [15]. This might make them well positioned to advocate an ethical discussion on biobanking [1]. Our blood donors reported having learned to appreciate the importance of blood donation, while they saw themselves as being in a process of discussing and learning more about biobank principles and beneficiaries of which they expected to hear more from the BSB.

## Supplementary information

Supplementary Table S1

Supplementary Table S2

Supplementary Table S3
